# Spontaneous Control of SIV Replication Does Not Prevent T Cell Dysregulation and Bacterial Dissemination in Animals Co-Infected with M. tuberculosis

**DOI:** 10.1128/spectrum.01724-21

**Published:** 2022-04-25

**Authors:** Ryan V. Moriarty, Mark A. Rodgers, Amy L. Ellis, Alexis J. Balgeman, Erica C. Larson, Forrest Hopkins, Michael R. Chase, Pauline Maiello, Sarah M. Fortune, Charles A. Scanga, Shelby L. O’Connor

**Affiliations:** a Department of Pathology and Laboratory Medicine, University of Wisconsin-Madison, Madison, Wisconsin, USA; b Department of Microbiology and Molecular Genetics, University of Pittsburgh, Pittsburgh, Pennsylvania, USA; c Center for Vaccine Research, University of Pittsburgh, Pittsburgh, Pennsylvania, USA; d Department of Immunology and Infectious Diseases, Harvard T.H. Chan School of Public Health, Boston, Massachusetts, USA; Indian Institute of Science Bangalore

**Keywords:** MCM, Mtb, SIV, coinfection

## Abstract

Individuals co-infected with HIV and Mycobacterium tuberculosis (Mtb) are more likely to develop severe tuberculosis (TB) disease than HIV-naive individuals. To understand how a chronic pre-existing Simian immunodeficiency virus (SIV) infection impairs the early immune response to Mtb, we used the Mauritian cynomolgus macaque (MCM) model of SIV/Mtb co-infection. We examined the relationship between peripheral viral control and Mtb burden, Mtb dissemination, and T cell function between SIV+ spontaneous controllers, SIV+ non-controllers, and SIV-naive MCM who were challenged with a barcoded Mtb Erdman strain 6 months post-SIV infection and necropsied 6 weeks post-Mtb infection. Mycobacterial burden was highest in the SIV+ non-controllers in all assessed tissues. In lung granulomas, the frequency of TNF-α-producing CD4^+^ T cells was reduced in all SIV+ MCM, but IFNγ-producing CD4^+^ T cells were only lower in the SIV+ non-controllers. Further, while all SIV+ MCM had more PD1+ and TIGIT+ T cells in the lung granulomas relative to SIV-naive MCM, SIV+ controllers exhibited the highest frequency of cells expressing these markers. To measure the effect of SIV infection on within-host bacterial dissemination, we sequenced the molecular barcodes of Mtb present in each tissue and characterized the Mtb population complexity. While Mtb population complexity was not associated with SIV infection group, lymph nodes had increased complexity when compared with lung granulomas across all groups. These results provide evidence that SIV+ animals, independent of viral control, exhibit a dysregulated T cell immune response and enhanced dissemination of Mtb, likely contributing to the poor TB disease course across all SIV/Mtb co-infected animals.

**IMPORTANCE** HIV and TB remain significant global health issues, despite the availability of treatments. Individuals with HIV, including those who are virally suppressed, are at an increased risk to develop and succumb to severe TB disease when compared with HIV-naive individuals. Our study aims to understand the relationship between the extent of SIV replication, mycobacterial growth, and T cell function in the tissues of co-infected Mauritian cynomolgus macaques during the first 6 weeks of Mtb infection. Here we demonstrate that increased viral replication is associated with increased bacterial burden in the tissues and impaired T cell responses, and that the immunological damage attributed to virus infection is not fully eliminated when animals spontaneously control virus replication.

## INTRODUCTION

Human immunodeficiency virus (HIV) and tuberculosis (TB) remain significant global health burdens, despite the wide availability of treatment (1). Further, individuals infected with HIV are at increased risk of developing and succumbing to active TB disease, as well as reactivation of latent TB (2, 3), regardless of whether antiretroviral therapy (ART) has successfully controlled HIV viremia (4–6). However, the reasons underlying why HIV+ individuals exhibit increased susceptibility to TB disease, independent of their chronic viral load set point, remains a mystery.

The dual susceptibility of nonhuman primates (NHP) to both Simian immunodeficiency virus (SIV), an NHP analog of HIV, and TB has additionally allowed researchers to further probe how the immune system simultaneously combats these pathogens within individual tissues over time. Importantly, the utilization of animal models of co-infection has allowed researchers to control the dose, route, and timing of infection. Often, humans with both HIV and TB are not sure which pathogen they were infected with first or the time between initial infection and co-infection. While most NHP studies focus on how latent TB, which is the most common form of TB in humans, is reactivated by a newly acquired SIV infection (7–9), few focus on how a pre-existing SIV infection impacts the host’s ability to contain and clear a new Mtb infection (10, 11). In this study, we focus on the latter, in which macaques with a pre-existing SIV infection who either spontaneously controlled viral replication (SIV+ viral controllers) or maintained persistently high viral loads (SIV+ viral non-controllers) are co-infected with Mtb and compared them with an SIV-naive cohort.

In order to examine the effect of whether control of a pre-existing SIV infection impacted the ability to control a new Mtb infection, we utilized our previously developed Mauritian cynomolgus macaque (MCM) model of SIV and Mtb co-infection. MCMs have limited MHC genetic diversity, such that nearly all of their MHC alleles can be explained by seven common MHC haplotypes (M1 to M7) (12). Approximately half of MCM containing at least one M1 MHC haplotype spontaneously control viremia following SIVmac239 infection to ≤10^3^ viral copies/mL of plasma, which is considered standard in the SIV field for viral control (13, 14).

We originally found that all M1+ MCM infected with SIV for 6 months followed by Mtb infection for up to 12 weeks developed rapidly progressive TB disease, independent of whether they were spontaneous SIV+ viral controllers (10). In this same study, we observed an increase in the number of granulomas in SIV+ animals between 4 and 8 weeks post-Mtb infection when compared with SIV-naive animals (10). These results implied that collecting granulomas at 6 weeks after infection with Mtb may provide a snapshot of the extent of local SIV and Mtb burden, as well as the corresponding T cell response, prior to the development of pathological TB disease. As a result, we subsequently explored TB disease in SIV+ and SIV-naive MCMs who were euthanized at 6 weeks post-Mtb infection (15), and we found that SIV+ animals had a lower CD4:CD8 ratio and increased immune activation in the Mtb-affected tissues when compared to those who were SIV-naive. In that study, however, we did not explore whether the control of SIV replication was related to (i) the extent of Mtb replication or dissemination or (ii) the frequency of CD4^+^ and CD8^+^ T cells producing cytokines in the granulomas.

In the current study, we now dissect out the relationship between the extent of SIV replication and control of Mtb from this previous animal study. By categorizing the animals according to whether or not they spontaneously controlled SIV replication, we examine how the control of peripheral viremia impacted Mtb pathogenesis when compared to SIV-naive MCMs. Because these animals were also infected with a molecularly barcoded Mtb strain, we can examine the extent to which bacterial dissemination was associated with the level of peripheral SIV replication. Lastly, we assess whether increased SIV replication influenced the immunological phenotype of total CD4^+^ and CD8^+^ T cells in lung tissue, thoracic lymph nodes (LN), and affected lung and LN granulomas. Cumulatively, this study elaborates on the highly complex system in which the extent of SIV replication is insufficient to predict the impact on TB disease and immunity.

## RESULTS

### Control of plasma SIV replication mirrors that observed in tissues of SIV/Mtb co-infected animals.

**FIG 1:**
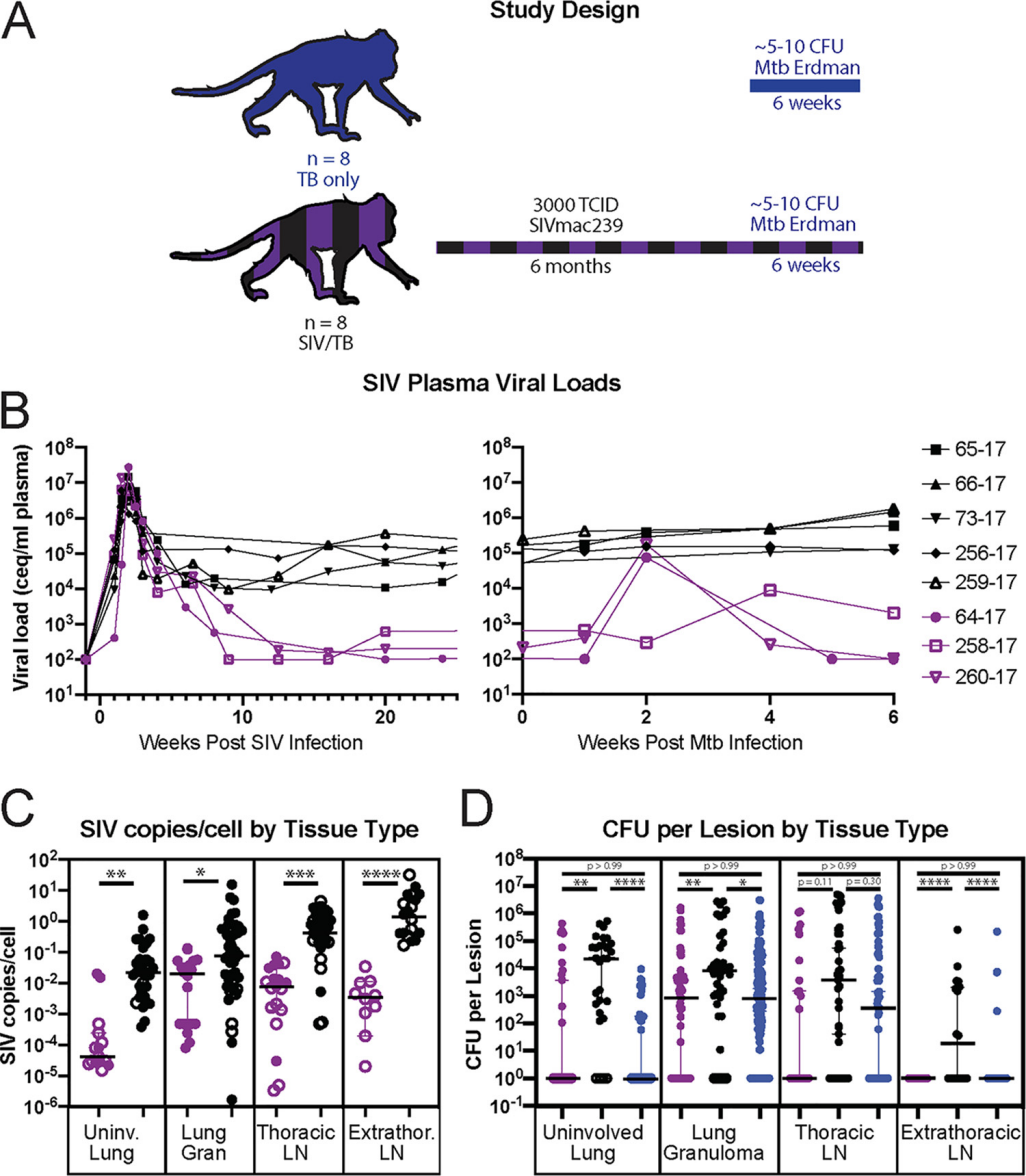
(A) Study design. (B) SIV plasma viral loads in our eight SIV/TB co-infected MCM. SIV plasma viral loads were used to determine viral controller status, with purple indicating animals with a viral load set point less than or equal to 10^4^ SIV copies/mL of plasma, and black indicating animals with a viral load set point greater than 10^4^ SIV copies/mL of plasma. Each animal is designated with a unique color/shape combination. (C) SIV copies per cell in uninvolved lung (leftmost panel), lung granuloma (second from the left), thoracic lymph node (second from the right), and extrathoracic lymph node (rightmost panel) samples for SIV viral controllers (purple) and SIV viral non-controllers. Each dot indicates an individual sample, with open circles indicating CFU- (sterile) samples. (D) CFU + 1 per lesion by tissue type for SIV viral controllers (purple), SIV viral non-controllers (black), and TB-only (SIV-naive, blue) animals. Each dot indicates an individual sample. CFU counts were transformed by adding 1 to accommodate sterile samples. Lines represent median +/– 95% CI. Significance determined by Kruskal-Wallis with Dunn’s test for multiple comparisons: *, *P* < 0.05; **, *P* <0.01; ***, *P* < 0.001; and ****, *P* < 0.0001.

For this analysis, we used samples derived from a previously reported animal study (15, 16). One cohort of 8 M1+ MCM was infected with SIVmac239 for 6 months, followed by co-infection with a low dose (3 to 19 CFU) of a molecularly barcoded Mtb Erdman strain for 6 weeks (Fig. 1A). A second cohort of 8 SIV-naive MCM were infected with the same strain and dose of Mtb for 6 weeks (15, 16). There were no significant differences in CFU dose between cohorts (Fig. S1A) or the extent of gross pathology as measured by necropsy scores (data not shown). The clinical outcomes and more comprehensive T cell analyses of this study are further described in Larson et al. (15) and Ellis et al. (16). Animals were necropsied 6 weeks after Mtb infection to characterize the association of the extent of SIV replication with antimycobacterial T cell responses in tissues prior to the development of severe TB pathology (e.g., TB pneumonia) (10). Prior to necropsy, a PET-CT scan is completed to guide excision of Mtb-affected tissues; additional lung granulomas identified during necropsy are also excised and analyzed. Flow cytometry is performed on all fresh samples, as cells are available (15).

SIV plasma viremia was characterized in the SIV-infected cohort (Fig. 1B). All animals had at least one copy of the M1 MHC haplotype (Table S1), so we expected approximately half the animals would spontaneously control SIV (14). We found that three SIV+ animals spontaneously achieved a plasma viral load set point of ≤10^3^ viral copies/mL, and were considered SIV+ viral controllers, as ≤10^4^ viral copies/mL is a common standard threshold for control in the field of HIV/SIV (14) (Fig. 1B, purple). The other five animals had a set point of at least 10^4^ copies/mL, which we considered SIV+ viral non-controllers (Fig. 1B, black). 2 weeks after the SIV+ animals were co-infected with Mtb, we detected a transient 2 to 3 log_10_ spike in viremia in two of the three SIV+ controllers, but not the SIV+ non-controllers (Fig. 1B). The plasma viremia in these animals spontaneously returned to baseline by 5 weeks post-Mtb infection. This observation is a unique example of how co-infection of an SIV+ individual with a secondary pathogen, such as Mtb, can induce transient virus replication. This type of transient SIV replication followed by immune-mediated virus control typically goes undetected in co-infected individuals.

Six weeks post-Mtb infection, necropsies were performed and individual granulomas, thoracic and extra-thoracic LN, and uninvolved lung tissue were collected and homogenized. Lung tissue was considered “uninvolved” if it did not exhibit macroscopic signs of TB disease at the time of necropsy. However, as these samples may harbor Mtb bacilli without overt lesions, all lung tissue samples were plated for bacterial burden measured by CFU. To assess the viral burden within each sample, tissues extracted during necropsy were homogenized, placed in TRIzol, and viral RNA was isolated as described in the methods. SIV *gag* copies/mL were measured in viral RNA from tissue homogenates by qPCR, and SIV copies/cell were calculated as described in the methods. As expected, we found that SIV+ viral controllers had fewer SIV copies/cell in the majority of their tissue samples compared with SIV+ viral non-controllers (Fig. 1C). Importantly, because individual tissue samples from a single animal are not truly independent and samples from one animal may overly bias the results, we also compared the median SIV copies/cell per animal between SIV+ viral controllers and viral non-controllers as a more statistically robust measure of viral burden between infection groups. With this approach, we also found that SIV+ controllers had a lower median viral burden than non-controllers, but this was not statistically significant with so few total animals (Fig. S2A).

### Poor control of plasma viremia is associated with increased Mtb bacterial burden in tissues.

We compared the bacterial burden in lung tissue, lung granulomas, and LN between SIV+ viral controllers, viral non-controllers, and SIV-naive MCM. We hypothesized that the SIV+ controllers (Fig. 1D, purple) and SIV-naive (Fig. 1D, blue) MCM would have similar Mtb bacterial burdens, while the SIV+ viral non-controllers (Fig. 1D, black) would have higher bacterial burden compared with the other cohorts. We found no statistically significant differences in Mtb burden between the SIV+ viral controllers and the SIV-naive MCM for any of the tissues examined (Fig. 1D, purple versus blue). In contrast, the bacterial burden was higher in lung tissue, lung granulomas, and extra-thoracic LN in the SIV+ viral non-controllers (black) than in the SIV-naive (blue) and SIV+ viral controller (purple) animals (Fig. 1D). Again, we compared the animal median CFU in each tissue type to confirm that a single animal was not overly biasing our lesion-based analysis. While we observed a similar pattern to the lesion-based analysis, the only significant relationship was between the lung granulomas of SIV+ viral non-controllers when compared with the SIV-naive animals. (Fig. S2B).

We then compared the total Mtb bacterial burden per animal across all three groups. In contrast to our analysis of individual lesions, the total thoracic bacterial burden, which includes CFU counts from uninvolved lung tissue, lung granulomas, and lymph nodes, was no different across groups (Fig. S3A), similar to what was observed when SIV+ MCM were considered as a single group (15). We also compared the frequency of sterile (CFU–) lesions between the groups. Regardless of if SIV+ MCM were stratified by controller status, there were no statistically significant differences between the percent of sterile granulomas in SIV-infected MCM when compared to those who were SIV-naive (Fig. S3B). This suggests that control of peripheral viremia is not related to the percentage of sterile granulomas present at 6 weeks following Mtb co-infection.

We then examined if there was a correlation between bacterial burden and viral burden in the same tissues across all SIV+ animals. With the limited numbers of animals and samples, we could not stratify these correlations by tissue type or SIV controller status. As a single group, we found a significant positive correlation between bacterial burden and the amount of virus per cell in individual tissues (Fig. S4). Taken together with our results from Fig. 1, we posit that the presence of actively replicating SIV in a given tissue impairs host control of mycobacterial growth, leading to an overall increase in total tissue CFU.

### SIV+ viral non-controllers have lower CD4^+^ T cell frequencies in both lung granulomas and thoracic LN when compared with SIV+ viral controllers or SIV-naive animals.

**FIG 2:**
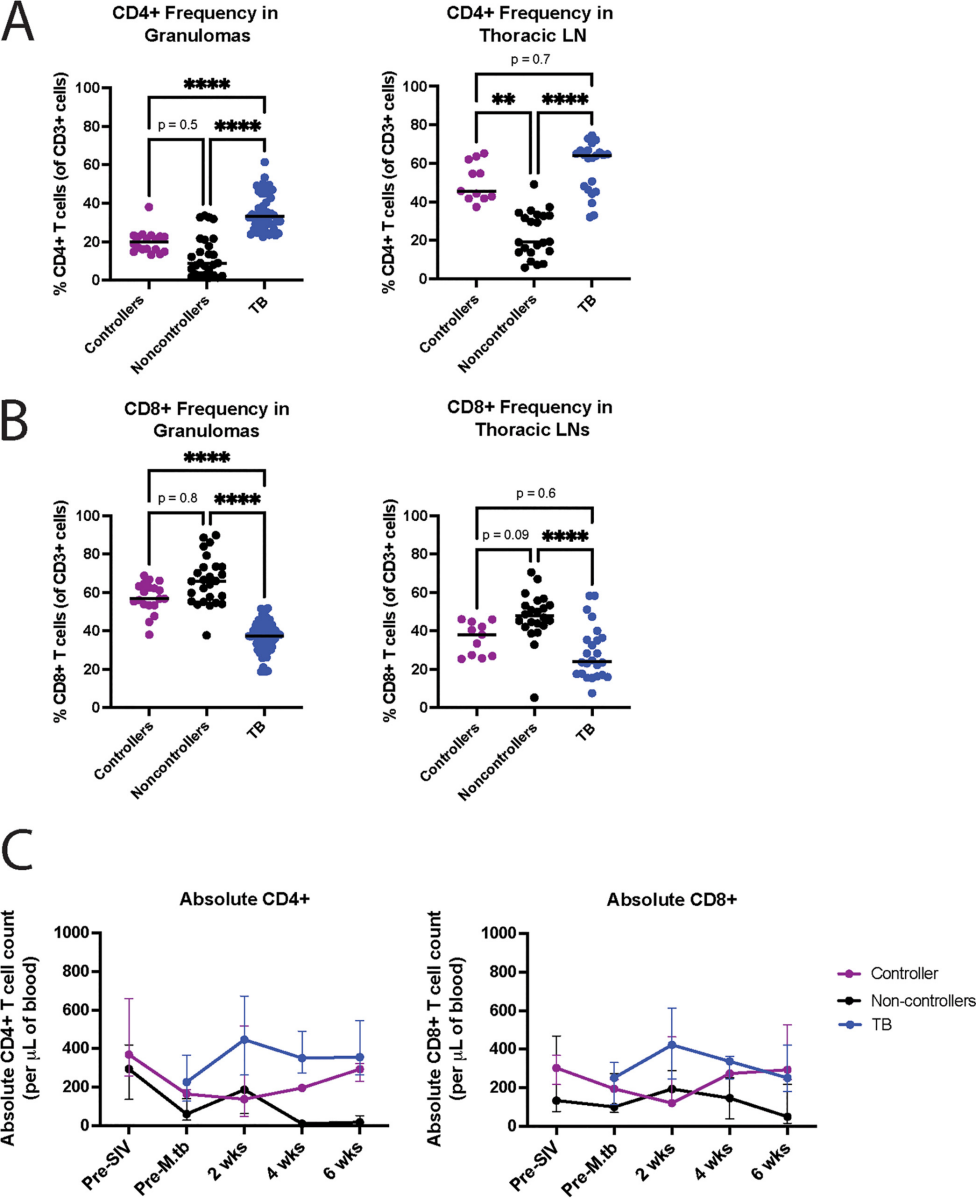
Frequency of total CD4^+^ (A) and total CD8^+^ (B) T cells in lung granulomas (left) and thoracic lymph nodes (right) of spontaneous SIV viral controllers (purple), SIV viral non-controllers (black), and TB-only (SIV-naive, blue) animals. Each dot indicates an individual lung granuloma (left) or thoracic LN (right) sample. Line represents the median. Significance determined by Kruskal-Wallis with Dunn’s test for multiple comparisons: *, *P* <0.05; **, *P* < 0.01; ***, *P* < 0.001; ****, *P* < 0.0001. (C) Absolute counts of total CD4^+^ (left) and total CD8^+^ (right) T cells isolated from peripheral blood prior to SIV infection, prior to Mtb infection, and every 2 weeks post-Mtb infection until necropsy. SIV viral controller cohort (*n* = 8) shown in purple, SIV viral non-controller cohort (*n* = 5) shown in black, and TB-only (SIV-naive) cohort (*n* = 8) shown in blue. Dots represent the median value for each group at a time point, and error bars indicate the interquartile range.

We first examined the frequency of CD4^+^ and CD8^+^ T cells in granulomas and thoracic LNs separately for SIV+ viral controllers and viral non-controllers, as all SIV+ animals were grouped together previously (15). Even though the frequencies of CD4^+^ T cells were lower and CD8^+^ T cells were higher in the tissues of all the SIV+ animals, it was more pronounced in the viral non-controllers, but not always significantly different from the viral controllers (Fig. 2). Both SIV+ viral controllers and viral non-controllers had significantly lower CD4^+^ T cell frequencies in the granulomas when compared with the SIV-naive MCM (Fig. 2A). Within the thoracic LNs, there were significantly lower CD4^+^ T cell frequencies in the SIV+ viral non-controllers when compared to the SIV+ viral controllers and the SIV-naive animals (Fig. 2A), which also led to a corresponding increase in the frequency of CD8^+^ T cells. The frequency of CD8^+^ T cells was significantly higher in the granulomas of each independent SIV+ cohort when compared with the SIV-naive animals (Fig. 2B), but only the SIV+ viral non-controllers had a higher frequency of CD8^+^ T cells in the thoracic LNs when compared with the SIV-naive animals (Fig. 2B). This was likely attributed to trafficking of antiviral CD8^+^ T cells to sites of SIV replication.

All groups had similar absolute CD4^+^ and CD8^+^ T cell counts in the blood prior to Mtb infection. However, the SIV+ viral non-controllers had fewer T cells than the SIV+ viral controllers and the SIV-naive animals in the blood at 6 weeks post-Mtb infection (Fig. 2C). This is not surprising because the Mtb infection likely increased SIV replication and disrupted the T cell populations in the blood. There were no differences in the gross pathology at necropsy, suggesting that differences in these T cell counts in the blood did not impact TB pathology at this early time point (data not shown) (15).

### Lower frequencies of T cells producing IFNγ, but not TNF-α, in granuloma lesions of animals with uncontrolled SIV replication.

**FIG 3:**
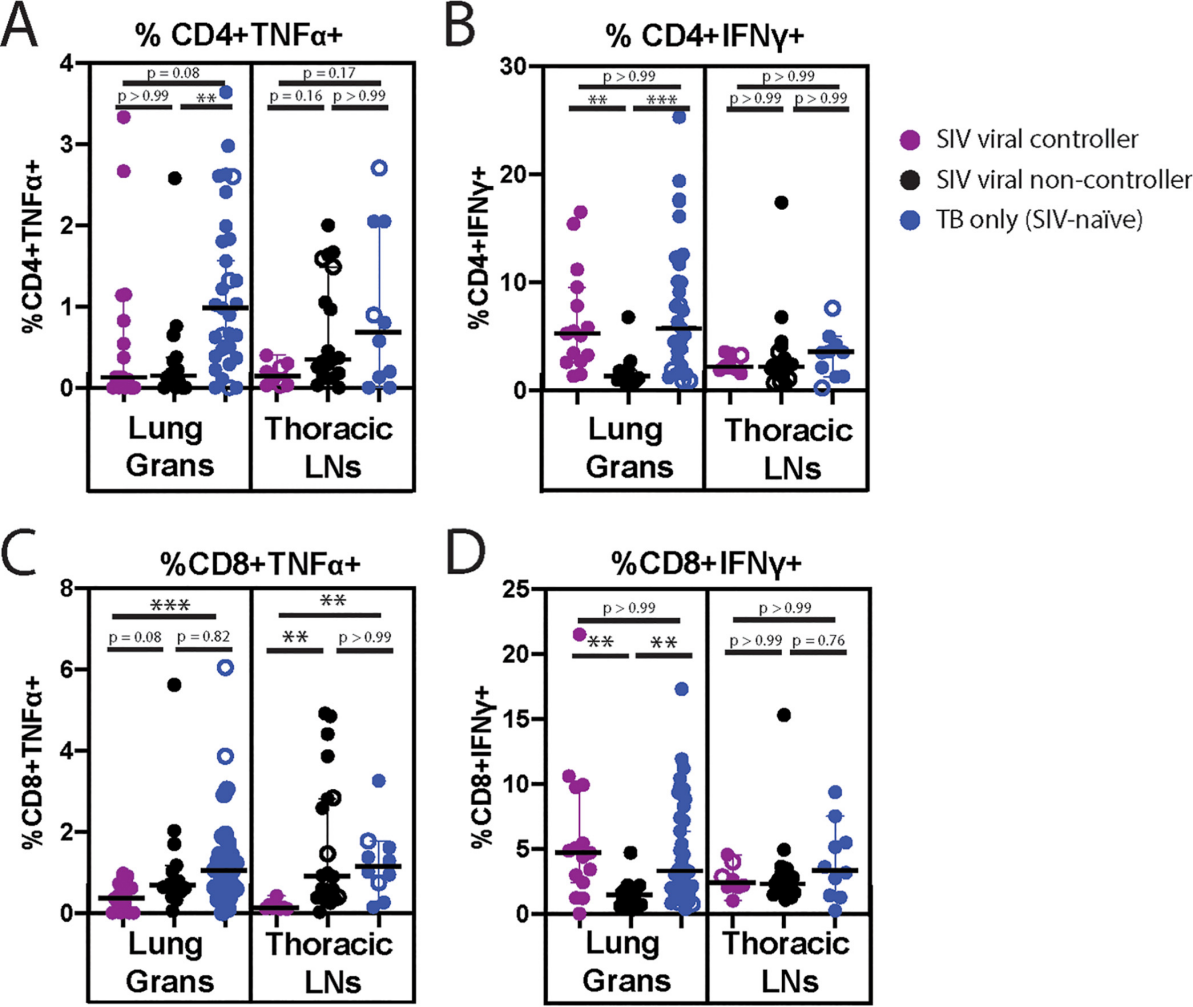
Frequency of total CD4^+^ (A, B) and total CD8^+^ (C, D) T cells producing TNF-α (A, C) or IFNγ (B, D) in lung granulomas (left panel) and thoracic LN (right panel) of spontaneous SIV+ viral controllers (purple), SIV+ viral non-controllers (black), and TB-only (SIV-naive, blue) animals. Each dot is an individual lung granuloma or thoracic LN sample. Open circles indicate sterile (CFU-) samples. Significance determined by Kruskal-Wallis with Dunn’s test for multiple comparisons: *, *P* < 0.05; **, *P* < 0.01; ***, *P* < 0.001; ****, *P* < 0.0001.

The frequency of CD4^+^ and CD8^+^ T cells that were producing the cytokines TNF-α- and IFNγ from lung granulomas, thoracic LNs, and lung tissue homogenates were measured as part of our previous study for the SIV+ group as a whole (15, 16). All tissue samples were examined *ex vivo* without external stimuli added. Even though the frequencies of total CD4^+^ and CD8^+^ T cells at necropsy were different for each group (Fig. 2), we also assessed whether the frequency of the T cells producing cytokines was impacted by the extent of SIV replication. We separated the SIV+ animals into viral controllers and viral non-controllers and compared the frequencies of cytokine-producing cells from each group with those in SIV-naive animals. Similar to Larson et al. (15), both groups of SIV+ animals, regardless of if they had controlled viral replication in the plasma, had a lower frequency of TNF-α-producing CD4^+^ cells when compared to SIV-naive animals (Fig. 3A). In contrast, although there was no detectable difference in the frequency of IFNγ-producing CD4^+^ T cells between SIV+ and SIV-naive animals (15), we observed distinct differences between SIV+ viral controllers and non-controllers. We found that the SIV+ viral non-controllers had the lowest frequency of IFNγ-producing CD4^+^ T cells in the lung granulomas, and this difference was significant when compared with both SIV viral controllers and TB-only (SIV-naive) animals (Fig. 3B). This key observation suggests that a greater extent of SIV replication is associated with a lower frequency of IFNγ-producing CD4^+^ T cells in lung granulomas.

We also performed a subgroup analysis of cytokine-producing CD8^+^ T cells. Similar to the analysis of CD4^+^ T cells, we found that the frequency of IFNγ-producing CD8^+^ T cells from the lung granulomas of SIV+ animals who did not control virus replication (SIV+ viral non-controllers) was significantly lower than either the SIV+ viral controllers or the TB-only (SIV-naive) animals (Fig. 3D). Surprisingly, the frequency of TNF-α-producing CD8^+^ T cells in the lung granulomas was lowest for the SIV+ viral controllers when compared with SIV+ viral non-controllers or those who were SIV-naive (Fig. 3C). This observation may be related to the antigen specificity of these CD8^+^ T cells (SIV vs Mtb-specific), which we were unable to explore at the time of necropsy.

We also examined the relationship between SIV disease and the frequencies of cytokine-producing T cells in the thoracic LNs, as LNs represent a major reservoir for HIV/SIV (17, 18). We found no difference in the frequency of *ex vivo* cytokine-producing CD4^+^ T cells in the thoracic LN between SIV+ groups, similar to studies of LNs isolated from HIV/Mtb co-infected individuals (19). Similar to the lesions, we found that the frequency of CD8^+^ T cells producing TNF-α in the thoracic LNs was lowest for the SIV+ viral controllers.

Again, recognizing there can be animal bias when considering all the lesions, we compared the animal medians for each of these immunological phenotypes. Unfortunately, with so few animals per group, all observed differences in the frequency of cytokine-producing T cells were only apparent when examining individual lesions or LNs, but not animal medians (Fig. S5).

These functional assays were performed without additional *ex vivo* peptide stimulation, so we do not know if the lesion- or LN-associated T cells were specific for SIV or Mtb. However, these data indicate that the extent of SIV control contributes to the cytokine milieu in the lung microenvironment when compared with TB-only (SIV-naive) animals. Further studies will be required to fully elucidate the relationships between the frequency of cytokine producing antigen-specific T cells in the tissues, localized Mtb replication, and systemic control of SIV.

### High frequencies of T cells expressing PD1 and TIGIT were present in the lung granulomas of SIV+ animals, independent of SIV viral controller status.

**FIG 4:**
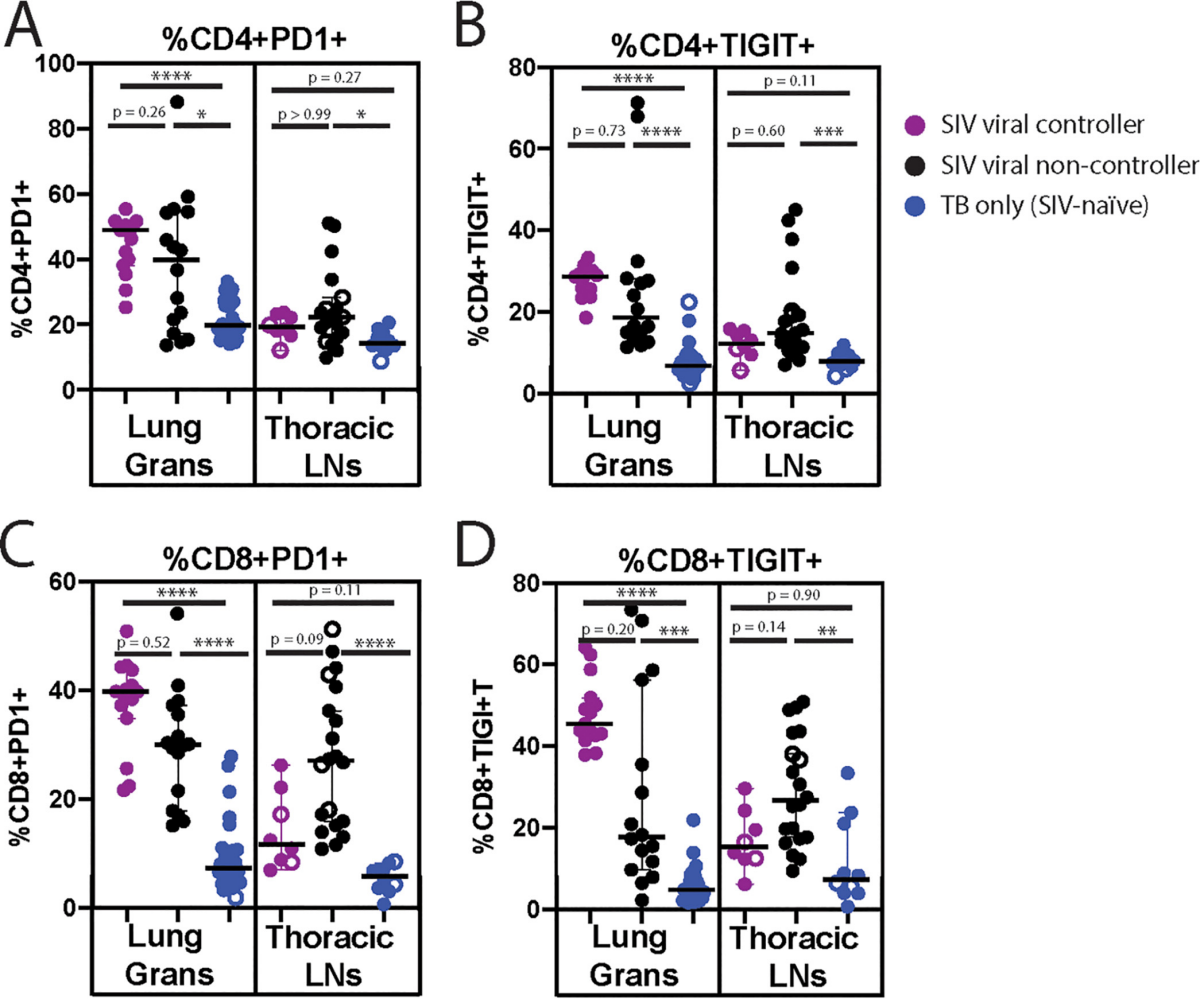
Frequency of total CD4^+^ (A, B) and total CD8^+^ (C, D) T cells expressing activation markers PD1 (A, C) and TIGIT (B, D) in the lung granulomas (left panel) and thoracic LN (right panel). Spontaneous SIV+ viral controllers are shown in purple, SIV+ viral non-controllers are shown in black, and TB-only (SIV-naive) animals are shown in blue. Each dot is an individual lung granuloma or thoracic LN sample. Open circles indicate CFU- samples. Significance is determined by Kruskal-Wallis with Dunn’s test for multiple comparisons: *, *P* < 0.05; **, *P* < 0.01; ***, *P* < 0.001; ****, *P* < 0.0001.

Similar to the analysis of cytokine-producing T cells, we sub-grouped animals into SIV+ viral controllers and viral non-controllers and examined the frequency of T cells expressing PD1 or TIGIT. In the lung granulomas, we found that both SIV+ viral controllers and viral non-controllers had a higher frequency of CD4^+^ and CD8^+^ T cells expressing PD1 or TIGIT when compared with TB-only (SIV-naive) animals. In the LNs, the frequency of CD4^+^ and CD8^+^ T cells expressing PD1 or TIGIT was highest in animals with uncontrolled SIV replication (Fig. 4). The LN results were not entirely surprising, as HIV replication, and by extension, SIV replication, occurs largely in the LN (17, 20). Further, PD1+ CD4^+^ T cells in the LN have been reported to be the primary source of replication-competent HIV (17) and correlate with viral load (21).

Again, we compared the median frequencies of the PD1+ and TIGIT+ T cell populations in each animal by combining the data from each lesion per animal (Fig. S6). Similar to the analysis of cytokine-producing T cells, differences were less significant, but the trends still existed.

### Thoracic lymph nodes are sites of increased Mtb dissemination.

Many prior reports describe increased extra-thoracic bacterial disease in individuals with severe HIV or SIV disease (11, 22, 23). Therefore, we hypothesized that actively replicating virus in a persistent HIV/SIV infection impairs the host’s ability to control bacterial growth and limit dissemination. We identified extrapulmonary granulomas in 13 of 16 animals, with one animal from each cohort lacking detectable granulomas (Table S2). Only one extrapulmonary tissue of an SIV+ viral controller had more than three granulomas detected. In contrast, the four SIV+ viral non-controllers had greater than 10 granulomas detected in at least one extrapulmonary tissue, with most tissues containing over 20 detectable granulomas. While there was a degree of variability within the SIV-naive cohort, these animals typically also had fewer granulomas in extrapulmonary tissues than the SIV+ viral non-controllers. These results suggest that uncontrolled SIV replication is associated with a greater number of visible granulomas in extrapulmonary tissue 6 weeks post-Mtb infection.

As part of this animal study, we included a molecularly barcoded Mtb Erdman strain (24), which allowed us to identify the distribution of the uniquely tagged bacteria throughout the thoracic cavity, including the uninvolved lung, LN, and granuloma sample across cohorts at the time of necropsy. This system was previously used in Chinese cynomolgus macaques to show that, in the absence of SIV, granulomas are typically seeded by a single bacterium and these lesions contribute to infection of the draining LN (24, 25). We used Simpson’s Diversity Index (SDI) and the number and frequency of unique barcoded Mtb in each sample to quantitatively examine the diversity and richness of the bacterial population present. With this metric, tissue samples containing more diverse populations of barcoded Mtb strains would have a value closer to 1, whereas samples with a single barcoded Mtb strain would have a value of 0. This allowed us to quantitatively assess how the presence and extent of SIV disease affected Mtb dissemination in the SIV/Mtb co-infection model.

**FIG 5:**
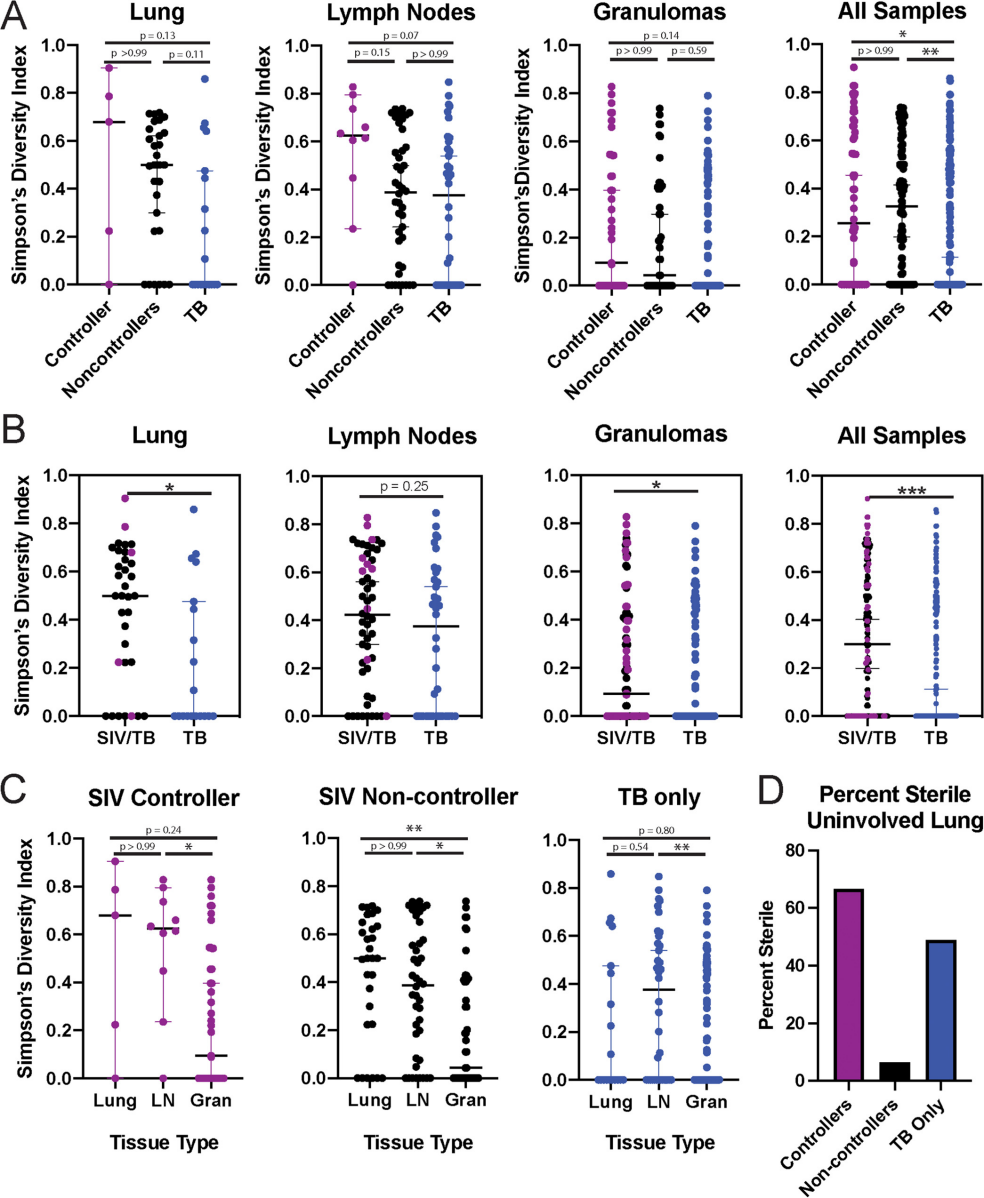
(A) Mtb barcode diversity as calculated by Simpson’s Diversity Index in uninvolved lung, lymph nodes (LN), lung granulomas, and all tissues combined, separated by infection and controller status, as well as (B) between SIV+ MCM and SIV-naive MCM. Each dot indicates an individual tissue sample. Lines represent median +/– 95% CI. (C) Mtb barcode diversity within each cohort. (D) Percent sterile uninvolved lung samples. SIV+ viral controllers are indicated in purple, SIV+ viral non-controllers in black, and TB-only (SIV-naive) in blue. Significance is determined by Kruskal-Wallis with Dunn’s test for multiple comparisons: *, *P* < 0.05; **, *P* < 0.01; ***, *P* < 0.001; ****, *P* < 0.0001.

Strikingly, we did not identify significant differences in the SDI of the Mtb population in individual tissues as a function of SIV control (Fig. 5A), but found that TB-only (SIV-naive) MCM had a lower SDI of the Mtb population in all tissues combined (indicating more lesions with a single barcoded Mtb strain) when compared with SIV+ MCM. When the SIV+ animals were combined into one group, the SDI of the Mtb population isolated from SIV+ MCM was significantly higher when compared with TB-only (SIV-naive) MCM, except for the thoracic LNs (Fig. 5B). Indeed, irrespective of SIV status, the SDI of the Mtb population found in the LN was significantly higher than in the lung granulomas (Fig. 5C). Notably, the SDI of the Mtb population isolated from lung samples that had no obvious disease at necropsy was only higher than in granulomas in the SIV+ viral non-controllers (Fig. 5C). This likely reflects greater bacterial dissemination in SIV+ viral non-controllers, because Mtb was more commonly found in tissues that had otherwise appeared uninvolved at the time of necropsy. In fact, we found that only 6% of uninvolved lung tissue was sterile in the SIV+ viral non-controllers, but 66 and 49% of uninvolved lung tissue was sterile in the SIV+ viral controllers and TB-only (SIV-naive) animals, respectively (Fig. 5D). These data support the hypothesis all MCMs have extensive Mtb replication in the LNs, but that the presence of an ongoing uncontrolled SIV infection increases mycobacterial dissemination in the lung granulomas and surrounding lung tissue.

## DISCUSSION

HIV/SIV+ individuals that control viral replication, either spontaneously or with antiretroviral treatment, typically exhibit less immune dysfunction when compared with individuals with uncontrolled HIV/SIV replication (26, 27). Similarly, it has been observed that antiretroviral treatment of HIV+ individuals reduces the TB incidence rate by up to 80% (28, 29). Unfortunately, human studies cannot dissect the underlying host-pathogen interactions that impact how the extent of HIV replication determines the trajectory of TB.

In the current study, we explore the relationship between control of SIV replication and TB disease in Mauritian cynomolgus macaques (MCMs). We use samples from a previous study that examined the disease pathology and immunology of SIV+ and TB-only (SIV-naive) animals at 6 weeks postinfection with M. tuberculosis (15, 16). In contrast to the previous assessment of all SIV+ animals as a single cohort, we examined features of the CD4^+^ and CD8^+^ T cell response that were restricted to either the SIV+ viral controller or viral non-controller animals and may have impacted TB disease 6 weeks post-Mtb infection or co-infection. At this early time point, we could characterize immune responses at the approximate initiation of the adaptive immune response (30, 31) but prior to when we could distinguish between active and latent TB. While it is possible that a proportion of the SIV-naive animals would have met the definition of latent TB infection eventually, the majority of animals had a high bacterial burden in lesions at the 6-week necropsy (Fig. 1D) (15), which is consistent with data from other studies of animals with active TB disease (25, 31).

We first examined whether local SIV replication correlated with the growth and dissemination of Mtb. We found a small but highly significant positive correlation between Mtb CFU and SIV copies/cell in Mtb-affected tissues across all the SIV+ animals (Fig. S4). This is consistent with other reports of increased bacterial burden in individual lung tissue, lung granulomas, thoracic LN, and extrapulmonary tissues of SIV/Mtb co-infected animals relative to those who were SIV-naive (10, 11). Even though we could not determine if SIV replication drove the growth of Mtb, or vice versa, this does provide additional evidence that the simultaneous presence of these pathogens may exacerbate the replication of both.

We also had the unique opportunity to observe that co-infection of SIV+ controllers with Mtb induced a detectable, but transient, spike in plasma SIV replication (Fig. 1B). It is possible that reactivation of latent virus from resting CD4^+^ T cells in the lung could contribute to this observed spike in plasma viremia (22, 23, 32, 33). Alternatively, indirect activation of T cells harboring SIV may contribute to this spike in plasma viremia, as the production of TNF-α associated with Mtb co-infection can contribute to increased viral replication (34–36). While we do not know the exact source of this transient virus spike, this data provides a nice example of how co-infection of SIV+ animals with a second pathogen transiently increases replication of SIV.

Because we used a barcoded Mtb Erdman strain, we could evaluate whether the extent of SIV infection affected the complexity of Mtb populations within the thoracic cavity, specifically, individual lung lesions, thoracic LNs, and lung tissue. While we detected granulomas in the spleen and/or liver of the majority of our animals (Table S2), we did not evaluate mycobacterial population diversity in these tissues, opting to focus on the thoracic cavity. In general, the complexity of the Mtb populations was similar between SIV+ controllers and non-controllers (Fig. 5A). As a group, SIV+ animals had tissues with more complex Mtb populations, when compared with TB-only (SIV-naive) animals (Fig. 5B), consistent with the hypothesis that SIV+ animals are less able to contain Mtb within individual lesions (37). This difference was largely driven by the complexity observed in the lung lesions and lung tissue, as both SIV+ and TB-only (SIV-naive) animals exhibited similar Mtb population complexity in the LNs. This was not surprising, as Mtb persists in the LNs (38), and a small number of LNs were found to have more complex Mtb populations in a separate study using this barcoded strain of Mtb (24). Even without measuring Mtb population diversity in the spleen and liver, the mere detection of Mtb at these extrapulmonary sites is consistent with bacterial dissemination that has been observed in HIV/SIV infected individuals (11). Future studies that focus on the collection and evaluation of Mtb in extrapulmonary tissues of SIV+ animals may prove helpful in understanding Mtb dissemination outside of the thoracic cavity.

One interesting set of tissues we studied was lung tissue lacking macroscopic evidence of disease at the time of necropsy, which we called “uninvolved lung.” In the SIV+ viral non-controllers, only 6% of these tissues were sterile. However, in the SIV+ viral controllers and TB-only (SIV-naive) animals, we found that 66% and 49%, respectively, were sterile (Fig. 5D). These data further supported our previous observation (10) that the number of granulomas dramatically increased in SIV+ animals between 4- and 8-weeks post-Mtb. By examining these tissues at 6-weeks post-Mtb infection in the current study, we detected these nascent granulomas and actually found that they had complex populations of Mtb. These observations suggest that the host failed to contain Mtb, potentially due to fewer CD4^+^ T cells, leading to rapid TB disease throughout the lung.

We also examined how the extent of SIV replication affected CD4^+^ and CD8^+^ T cell frequencies in lung granulomas. Due to limited available cells from granulomas, the difficulties disaggregating cells from these tissues, and the number of unique antibodies we could include in each flow cytometry panel, we chose to focus on the frequencies of CD4^+^ and CD8^+^ T cells producing IFNγ and TNF-α, which are cytokines commonly associated with host defense against TB (31, 39–42). By separating the SIV+ animals into viral controllers and viral non-controllers, we have now characterized (i) whether SIV and Mtb influence their corresponding pathogenesis and (ii) whether the extent of SIV replication impacts the frequency of functional CD4^+^ or CD8^+^ T cells in the tissues with TB disease.

Larson et al. previously evaluated the frequency of these cells, as well as the frequency of CD4^+^ and CD8^+^ T cells expressing the activation markers PD1 and TIGIT in the SIV+ group as a whole (15). As a single group, the SIV+ animals had a higher frequency of T cells with activation markers, a diminution of those producing TNF-α, and no difference in the frequency of those producing IFNγ. We thought it was important to determine if the extent of SIV replication affected the frequency of these populations of cytokine-producing immune cells in the tissues, in an effort to identify reasons why HIV+ people treated with antiretrovirals are still more susceptible to TB than those who are HIV-naive.

We identified a significantly lower frequency of CD4^+^ T cells in the thoracic LN of SIV+ viral non-controllers when compared with the SIV+ viral controllers and TB-only (SIV-naive) animals (Fig. 2), a difference that could not be observed when assessing the SIV+ group as a whole, and in a tissue that was not examined in Larson et al. (15). This decrease in CD4^+^ T cell frequency is not surprising, as SIV replication is most robust in the LN (17, 18, 20), which likely contributes to a reduced frequency of CD4^+^ T cells in this location. Thoracic LNs are also important sites of mycobacterial replication (37, 38). Because of the importance of CD4^+^ T cells in controlling mycobacterial infection (32, 43), it could be hypothesized that a reduced frequency of CD4^+^ T cells in this location could contribute to increased bacterial burden or mycobacterial dissemination. However, while there was a difference in the frequency of total CD4^+^ T cells in the LN of SIV+ viral controllers and SIV+ viral non-controllers, the frequencies of IFNγ-producing CD4^+^ T cells was equal between these groups (Fig. 3). Given the importance of IFNγ in containing mycobacterial infection (44, 45), this may have contributed to why we did not observe differences in CFU per lesion in the LN (Fig. 1D) or mycobacterial population diversity (Fig. 5) in this tissue type between SIV+ viral controllers and viral non-controllers.

All SIV+ animals had lower frequencies of total CD4^+^ T cells in the lung granulomas when compared with the TB-only (SIV-naive) animals (Fig. 2A). However, we found that the SIV+ viral non-controllers had a significantly lower frequency of CD4^+^ T cells that were producing IFNγ when compared with the SIV+ viral controllers and the TB-only (SIV-naive) animals (Fig. 3B). This is a key distinction from Larson et al. (15), in which the SIV+ cohort as a whole did not exhibit differences in the frequency of CD4^+^ T cells producing IFNγ in the lung granulomas when compared to TB-only (SIV-naive) animals. This significantly lower frequency of CD4^+^ T cells producing IFNγ may have contributed to the increased bacterial burden in the lung granulomas of SIV+ viral non-controllers when compared to both the SIV+ viral controllers and TB-only (SIV-naive) animals (Fig. 1D). While we were able to identify differences in frequencies of CD4^+^ T cells producing IFNγ, we were unable to assess if there were differences in the amount of IFNγ produced by these cells given the parameters of this study.

In contrast to the IFNγ results, both SIV+ viral controllers and viral non-controllers had fewer T cells producing TNF-α compared with TB-only (SIV-naive) animals, which may further explain why even those with systemic viral containment are more susceptible to TB when compared with those who are otherwise healthy. Previous reports have demonstrated that TNF-α is vital in the anti-TB response (34, 41). However, SIV infection has been reported to reduce TNF-α production by Mtb-specific cells (36). Therefore, SIV-mediated reductions in TNF-α production may be contributing to the increased exacerbation of mycobacterial disease in our co-infected animals. Even though the number of animals is small, these data provide some initial key observations, such that future studies focused on comparing the frequencies of cytokine-producing CD4^+^ T cells between SIV/HIV controllers and progressors may be key to defining effective anti-mycobacterial immunity.

Cumulatively, this study highlights the importance of understanding the interaction between SIV and Mtb in tissues and the impact of increased viral replication on early TB disease. Because SIV+ viral controllers had limited peripheral viral replication, we hypothesize that spontaneous SIV+ viral controllers and ART-treated SIV+ MCM have similar immune responses to Mtb upon co-infection. Future studies will need to directly compare SIV-specific and Mtb-specific T cell responses in tissues of ART-treated and ART-naive SIV+ MCM using *ex vivo* functional assays to examine if tissues with reduced viral replication exhibit increased polyfunctional Mtb-specific T cells that can contribute to control of Mtb. Ultimately, the study reported here demonstrates that while spontaneous control of SIV replication can influence total bacterial burden in tissues and T cell phenotypes, this alone is not enough to explain differences in early development of TB disease in co-infected animals. Future studies will also need to evaluate how the extent of SIV replication affects chronic TB disease. Thus, this study will continue to prompt new questions about tissue-specific responses in SIV+ or HIV+ individuals who are co-infected with Mtb.

## MATERIALS AND METHODS

### Animal care and ethics statement.

Mauritian cynomolgus macaques (Macaca fascicularis; MCM) were obtained from Bioculture, Ltd. (Mauritius). All MCM chosen for this study were selected by genotyping to have at least one copy of the M1 MHC haplotype (46) (Table S1). Animals (*n* = 16) were housed in a BSL2+ facility at the University of Pittsburgh (U. Pitt.) during SIV infection and transferred to a BSL3+ facility within the Regional Biocontainment Laboratory for Mtb infection. The U. Pitt. Institutional Animal Care and Use Committee (IACUC) approved all animal procedures and protocols and adheres to guidelines established in the Weatherall report (8th Edition) and the Guide for the Care and Use of Laboratory Animals. The studies described here were conducted under IACUC study protocols 18032418 and 15035401, which were reviewed and approved by the U. Pitt. U. Pitt follows national guidelines established in the Animal Welfare Act (7 U.S.C. Sections 2131–2159) and Guide for the Care and Use of Laboratory Animals (8th Edition) as mandated by the U.S. Public Health Service Polity. U. Pitt’s Animal Welfare Act Assurance Number is A3187-01.

Macaques were pair-housed at U. Pitt in caging measuring 4.3 square feet per animal and spaced to allow visual and tactile contact with neighboring conspecifics in rooms with autonomously controlled temperature, humidity, and lighting. Animals had *ad libitem* access to water. Macaques were fed biscuits formulated for nonhuman primates (NHP) twice daily, with additional fresh fruits, vegetables, or foraging mix provided at least 4 days/week. Our NHP enrichment specialist designed and oversaw a three-component enhanced enrichment plan in which species-specific behaviors are encouraged. Animals are provided with access to toys and manipulanda filled with food treats such as frozen fruit and peanut butter, which are rotated regularly. Foraging behaviors are stimulated with puzzle feeders, cardboard tubes containing small food items, and foraging boards placed in the cage. Interactions between cages are stimulated using adjustable mirrors accessible to the animals. Human and macaque interactions are encouraged and occur daily, adhering to established safety protocols. While performing tasks in the housing area, animal caretakers are encouraged to interact with the animals through talking and facial expressions. A strict schedule is followed for routine procedures such as feeding and cage cleaning to allow the animals to acclimate to a daily schedule. Auditory and visual stimulation is provided to all macaques through radios or TV/video equipment showing cartoons or other formats for children in the housing areas. Enrichment is rotated routinely so animals are not repetitively exposed to the same videos or radios. Appetite, attitude, activity level, hydration status, etc. were checked at least twice daily. Animals were monitored closely for evidence of disease (anorexia, lethargy, coughing, dyspnea, tachypnea, etc.) following SIV and/or Mtb infection. Physical exams, including weights, were regularly performed. Ketamine was used to sedate animals prior to all veterinary procedures (blood draws, etc.). Disease progression was monitored following Mtb infection by monthly PET/CT imaging (47). Animals were closely monitored for any signs of pain or distress by experienced veterinary technicians, and appropriate medication or supportive care was provided if necessary. Any animal considered to have advanced disease or intractable pain or distress from any cause was sedated with ketamine and then humanely euthanized using sodium pentobarbital (65 mg/kg, IV). A trained veterinary professional confirms death by lack of heartbeat and pupillary responses.

### SIV and Mtb infection of MCM.

SIV/Mtb co-infected animals (*n* = 8) were infected intrarectally with 3,000 TCID_50_ SIVmac239. Six months following SIV infection, animals were co-infected with a low dose (3 to 19 CFU) of a molecularly barcoded Mtb (Erdman strain) (24) via bronchoscopic instillation, as described previously (10). Animals in the TB-only control group (*n* = 8) were infected with barcoded Mtb in an identical manner. Clinical testing and PET/CT imaging was used to monitor TB progression (10). Animals were humanely euthanized 6 weeks following Mtb infection. Necropsies were performed using PET/CT images to map individual granulomas and guide their excision. Random lung lobe samples, peripheral LN, and thoracic LN were also harvested (48); further described in Larson et al. (15).

### Sample collection.

Tissues collected at necropsy were homogenized using Medimachines (BD Biosciences) and an aliquot of each homogenate was plated on 7H11 agar plates to quantify Mtb CFU as previously described (10, 48). Another aliquot was used for flow cytometry, with viable cells quantified by trypan blue exclusion in a hemocytometer, as described in Larson et al. (15).

### Flow cytometry.

Flow cytometry was conducted as described previously (15, 16). All immunology data was re-analyzed from Larson et al. (15), stratifying the SIV+ animals into spontaneous SIV+ controllers or SIV+ non-controllers. Briefly, 1 × 10^6^ cells (or all cells obtained from granulomas with <1 × 10^6^ cells) were stained with 0.25 μg of Mamu MR1 5-OP-RU or Ac-6-FP tetramer for 1 h in the presence of 500 nM Dasatinib (Thermo Fisher Scientific; Cat No. NC0897653). When TCRVα7.2 co-staining was performed, the antibody was added 30 min after the addition of the MR1 tetramer. Cells were washed once with FACS buffer (10% fetal bovine serum [FBS] in a 1X PBS solution) supplemented with 500 nM Dasatinib, then surface antibody staining was performed for 20 min in FACS buffer + 500 nM Dasatinib. Antibodies used for surface staining are listed in Table 1. Samples were fixed in 1% paraformaldehyde for a minimum of 20 min. For intracellular staining, cells were washed twice with FACS buffer and staining with antibodies was performed in Medium B permeabilization buffer (Thermo Fisher Scientific, Cat. No. GAS002S-100) for 20 min at room temperature. For intranuclear staining, the True Nuclear Transcription Factor Buffer Set (Biolegend; San Diego, CA) was used according to manufacturer’s instructions. Briefly, cells were fixed in the TrueNuclear fixation solution for 1 h, then washed 3 times with the permeabilization buffer. Cells were then stained with the transcription factors indicated in Table 1 at 4°C for 1 h, rinsed 3 times with permeabilization buffer, then resuspended in FACS buffer. Flow cytometry was performed on a BD LSR II (Becton, Dickinson; Franklin Lakes, NJ), and the data were analyzed using FlowJo software for Macintosh (version 9.9.3 or version 10.1).

**TABLE 1:** Antibodies used in staining panels for flow cytometry

Marker	Clone(s)	Fluorochrome(s)	Purpose of use	Surface/intracellular/intranuclear
CD45	D058-1283	BV786	Lineage	Surface
CD3	SP34-2	AF700, BV650	T cell marker	Surface
CD4	OKT4, L200	PE Cy7, BV510, BV711, BV786	T cell marker	Surface
CD8	RPAT8, SK1	PacBlue, BV510, BV605, BV711, BV786	T cell marker	Surface
CD206	19.2	PE Cy5	Macrophage exclusion	Surface
TCRVα7.2	3C10	BV421, BV605	MAIT cell marker	Surface
CCR5	J418F1	BV421	Chemokine/MAIT lung homing	Surface
CCR6	11A9	PE CF594, PE Cy7	Chemokine/gut homing	Surface
CD127	MB15-18C9	PE	IL-7 receptor	Surface
CXCR3	G025H7	PE Dazzle 594	Chemokine/MAIT lung homing	Surface
CCR7	FAB197F	FITC	Chemokine/lymph homing	Surface
CD28	CD28.2	APC, BV510	T cell memory	Surface
CD95	DX2	PE Cy5	T cell memory	Surface
T-Bet	4B10	BV605	Transcriptional marker	Intranuclear
Eomes	WD1928	PE Cy7	Transcriptional marker	Intranuclear
RORγT	AFKJS-9	PE	Transcriptional marker	Intranuclear
PLZF1	R17-809	PE CF594	Transcriptional marker	Intranuclear
Fox3P	150D	AF488, AF647	Transcriptional marker	Intranuclear
HLADR	G46-6	BV650	T cell activation	Surface
CD39	eBioA1 (A1)	FITC, PE Cy7	T cell activation	Surface
CD25	BC96, M-A251	PE, BV605	T cell activation	Surface
CD69	TP1.55.3	ECD	T cell activation	Surface
ki67	B56	AF647	T cell proliferation	Intracellular
TIGIT	MBSA43	FITC, PerCP EFluor710	T cell activation/exhaustion	Surface
PD1	EH12.2H7	BV605	T cell activation/exhaustion	Surface
IFNγ	4S.B3	FITC, BV510	Cytokine	Intracellular
TNFα	Mab11	AF700, PerCP Cy5.5	Cytokine	Intracellular
CD107a	H4A3	APC, BV605	Degranulation	Surface
LIVE/DEAD	–^*a*^	Near IR	Dead cell stain	--

a –, indicates that there is no clone for that antibody and that it is not a surface, intranuclear, or intracellular marker, since it is used to exclude dead cells.

### SIV viral RNA quantification.

Viral RNA was isolated from necropsy tissue homogenates by adding TRIzol and extracting RNA via a standard phenol-chloroform extraction. Viral RNA was quantified using a *gag* qPCR assay as previously described (49). Viable cell counts were determined from an aliquot of the same homogenate and used to calculate the SIV copies per cell. If the cell count was below the limit of detection of the hemocytometer, the limit of detection divided by two was used to approximate the value.

### Mtb barcode sequencing.

Mtb genomic DNA was isolated as previously described (24). Following gDNA isolation, samples were quantified and diluted to 10 ng/μL. Samples were then amplified twice using 2x Q5 Master Mix (New England BioLabs) and two unique primer sets, one to add a molecular counter, and one to add the Illumina TruSeq adapter sequences. Primer sequences can be found in Table S3. Samples were then sequenced on an Illumina MiSeq using a 2 × 150 kit and v2 chemistry. A computational pipeline courtesy of MRC and the Fortune lab was used to determine barcode sequences. Barcode diversity was then determined using Simpson’s Diversity index, and comparisons between groups were done using Kruskal-Wallis with Dunn’s test for multiple comparisons. All figures were generated using Prism 8 and Adobe Illustrator 2019.

### Statistical analysis.

All total CFU counts were transformed by adding 1 to account for sterile granulomas and LN tissues in log-scale graphs. Statistics were done using Prism 8. Comparisons between groups were done using either Mann-Whitney, for comparisons between two groups, or Kruskal-Wallis with Dunn’s test for multiple comparisons. Spearman correlation coefficients were calculated to determine relationships between variables.

### Data availability.

All sequence data generated for this study is publicly available on the Sequence Read Archive under accession number PRJNA768113.
