# Supplementary material for: Spontaneous Control of SIV Replication Does Not Prevent T Cell Dysregulation and Bacterial Dissemination in Animals Co-Infected with M. tuberculosis
Source: Microbiol Spectr. 2022 Apr 25;10(3):e01724-21. doi: 10.1128/spectrum.01724-21 (PMC9241861; doi:10.1128/spectrum.01724-21)
Supplement: SUPPLEMENTAL FILE 1 — Supplemental material. Download spectrum.01724-21-s001.pdf, PDF file, 1.4 MB [file spectrum.01724-21-s001.pdf]

S1

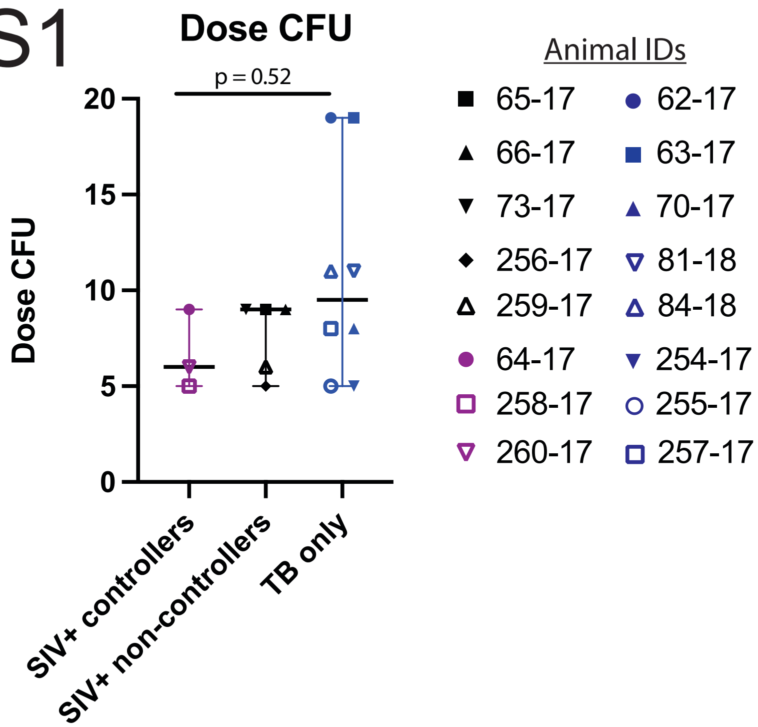

Dose CFU
p = 0.52
20
15
10
5
0
Dose CFU
SIV+ controllers
SIV+ non-controllers
TB only
Animal IDs
65-17
66-17
73-17
256-17
259-17
64-17
258-17
260-17
62-17
63-17
70-17
81-18
84-18
254-17
255-17
257-17

S2A

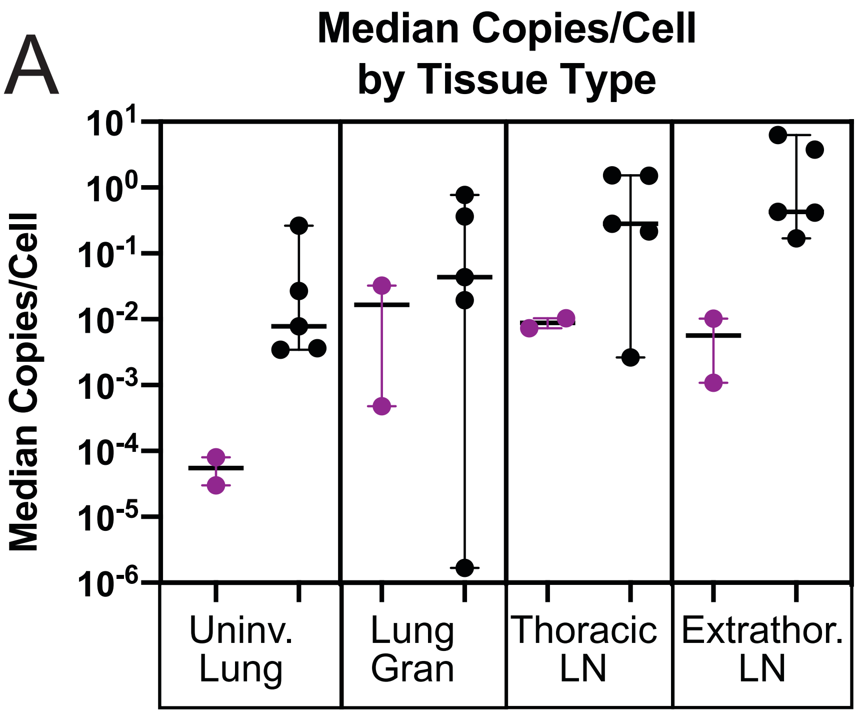


S2B

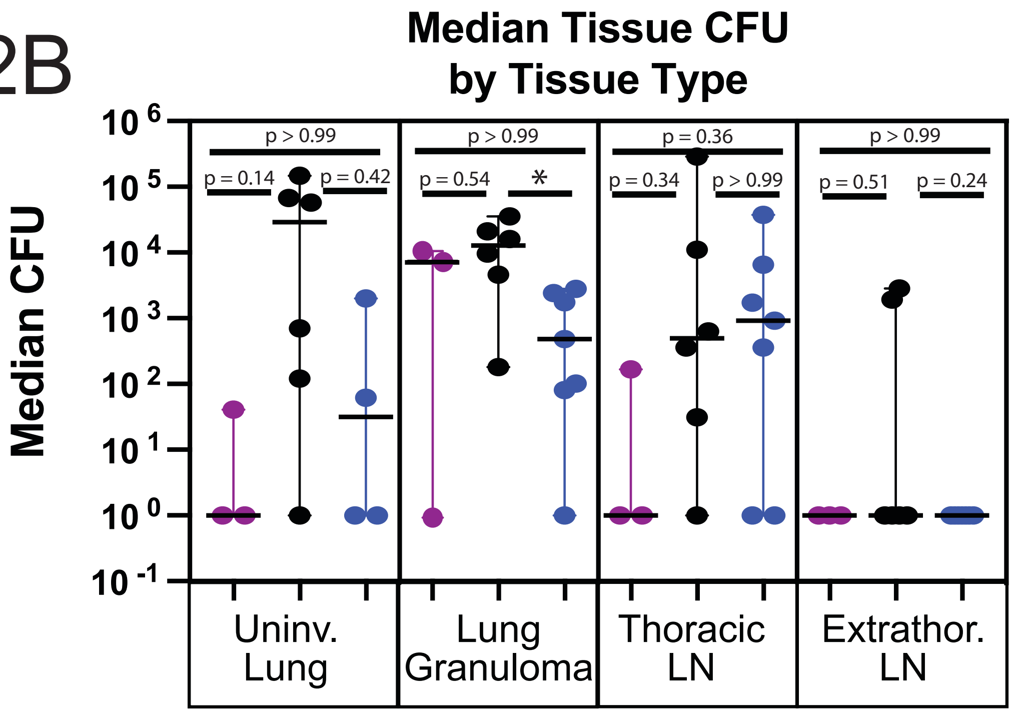


S3A

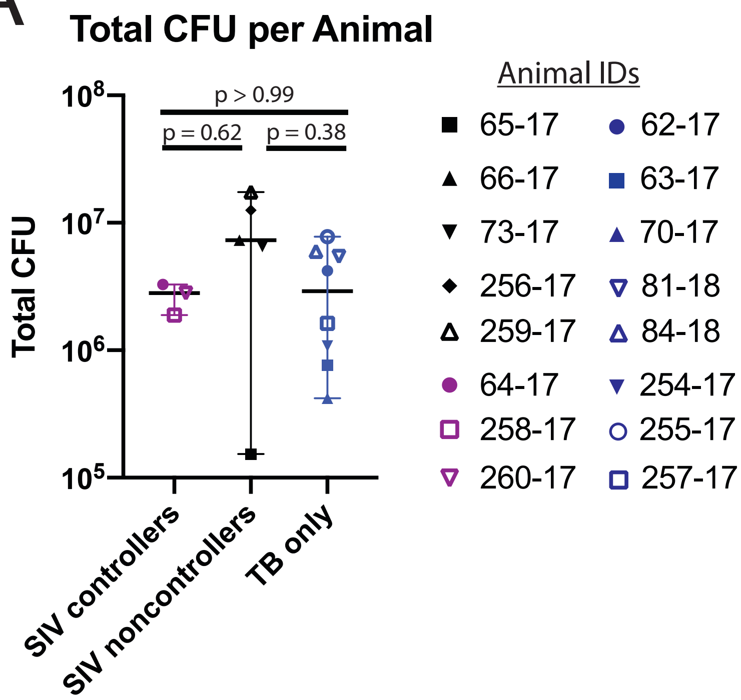


S3B

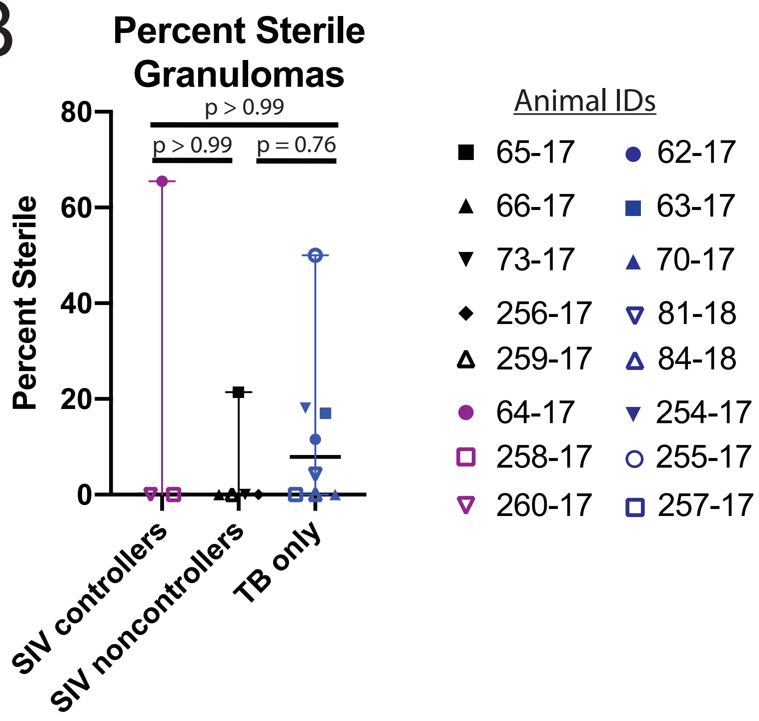

S4

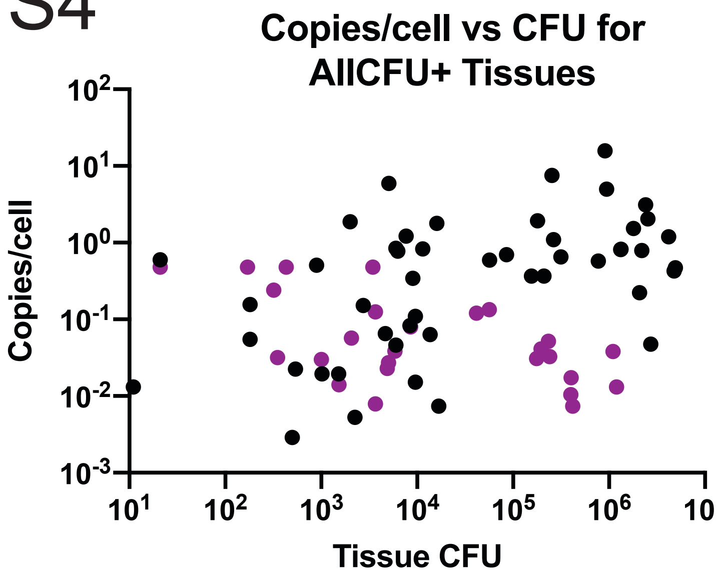


Spearman r = 0.3235 p = 0.0046

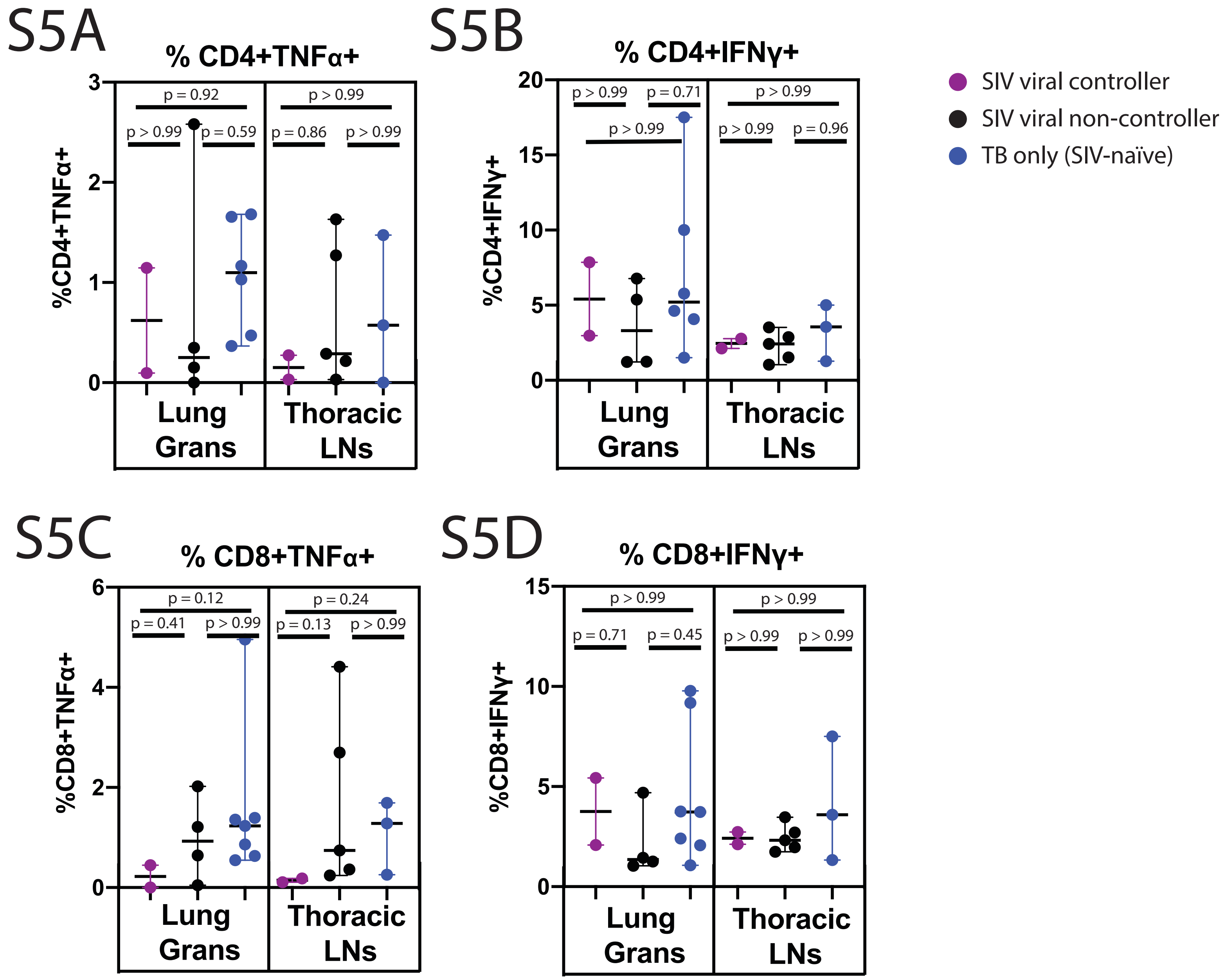

S5A
% CD4+TNFα+
%CD4+TNFα+
p = 0.92
p > 0.99
p = 0.59
p > 0.99
p = 0.86
p > 0.99
Lung Grans
Thoracic LNs
S5B
% CD4+IFNγ+
%CD4+IFNγ+
p > 0.99
p = 0.71
p > 0.99
p > 0.99
p > 0.99
p = 0.96
Lung Grans
Thoracic LNs
SIV viral controller
SIV viral non-controller
TB only (SIV-naïve)
S5C
% CD8+TNFα+
%CD8+TNFα+
p = 0.12
p = 0.41
p > 0.99
p = 0.24
p = 0.13
p > 0.99
Lung Grans
Thoracic LNs
S5D
% CD8+IFNγ+
%CD8+IFNγ+
p > 0.99
p = 0.71
p = 0.45
p > 0.99
p > 0.99
p > 0.99
Lung Grans
Thoracic LNs

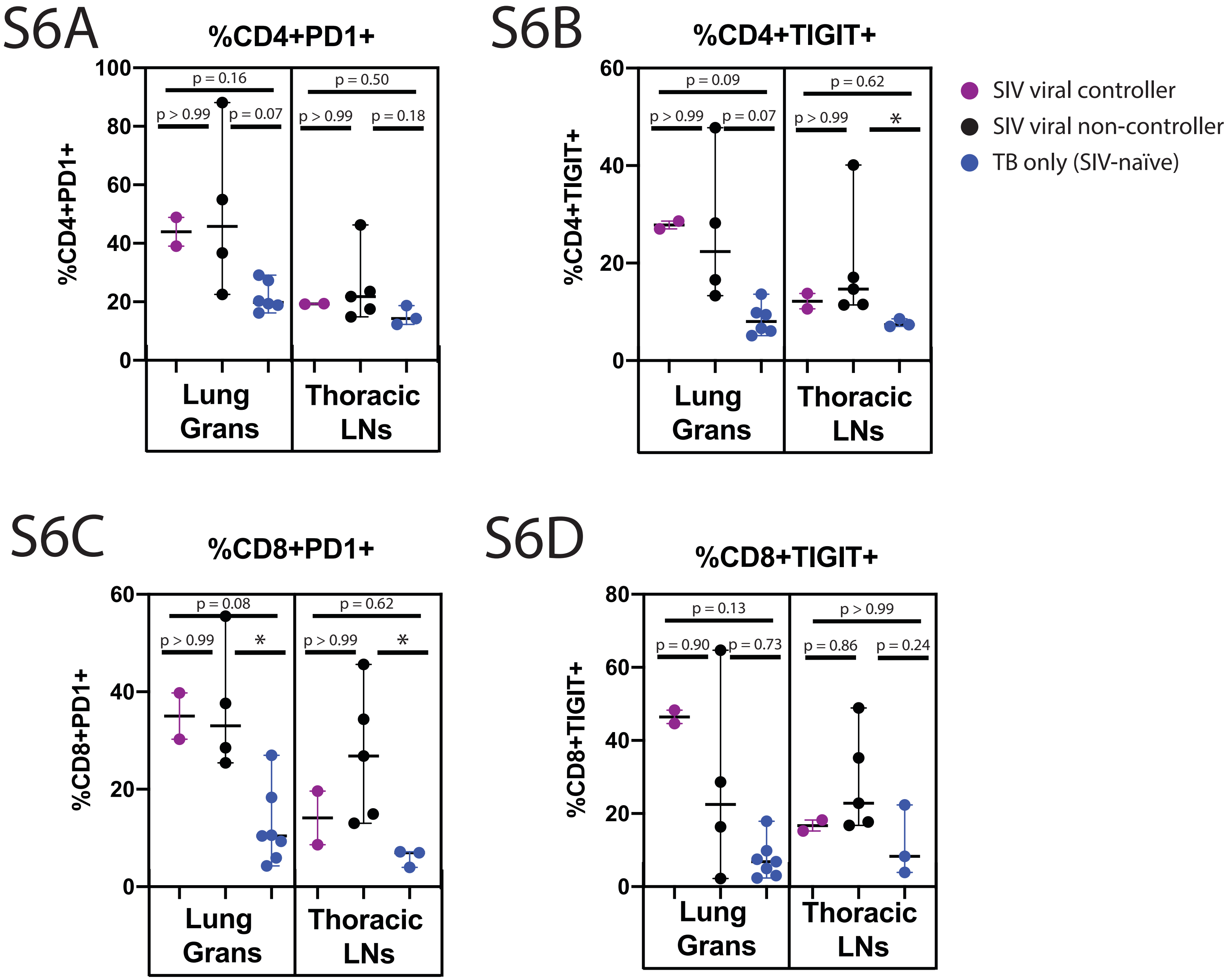
S6A
%CD4+PD1+
%CD4+PD1+
p = 0.16
p > 0.99
p = 0.07
p = 0.50
p > 0.99
p = 0.18
Lung Grans
Thoracic LNs
S6B
%CD4+TIGIT+
%CD4+TIGIT+
p = 0.09
p > 0.99
p = 0.07
p = 0.62
p > 0.99
*
Lung Grans
Thoracic LNs
SIV viral controller
SIV viral non-controller
TB only (SIV-naïve)
S6C
%CD8+PD1+
%CD8+PD1+
p = 0.08
p > 0.99
*
p = 0.62
p > 0.99
*
Lung Grans
Thoracic LNs
S6D
%CD8+TIGIT+
%CD8+TIGIT+
p = 0.13
p = 0.90
p = 0.73
p > 0.99
p = 0.86
p = 0.24
Lung Grans
Thoracic LNs

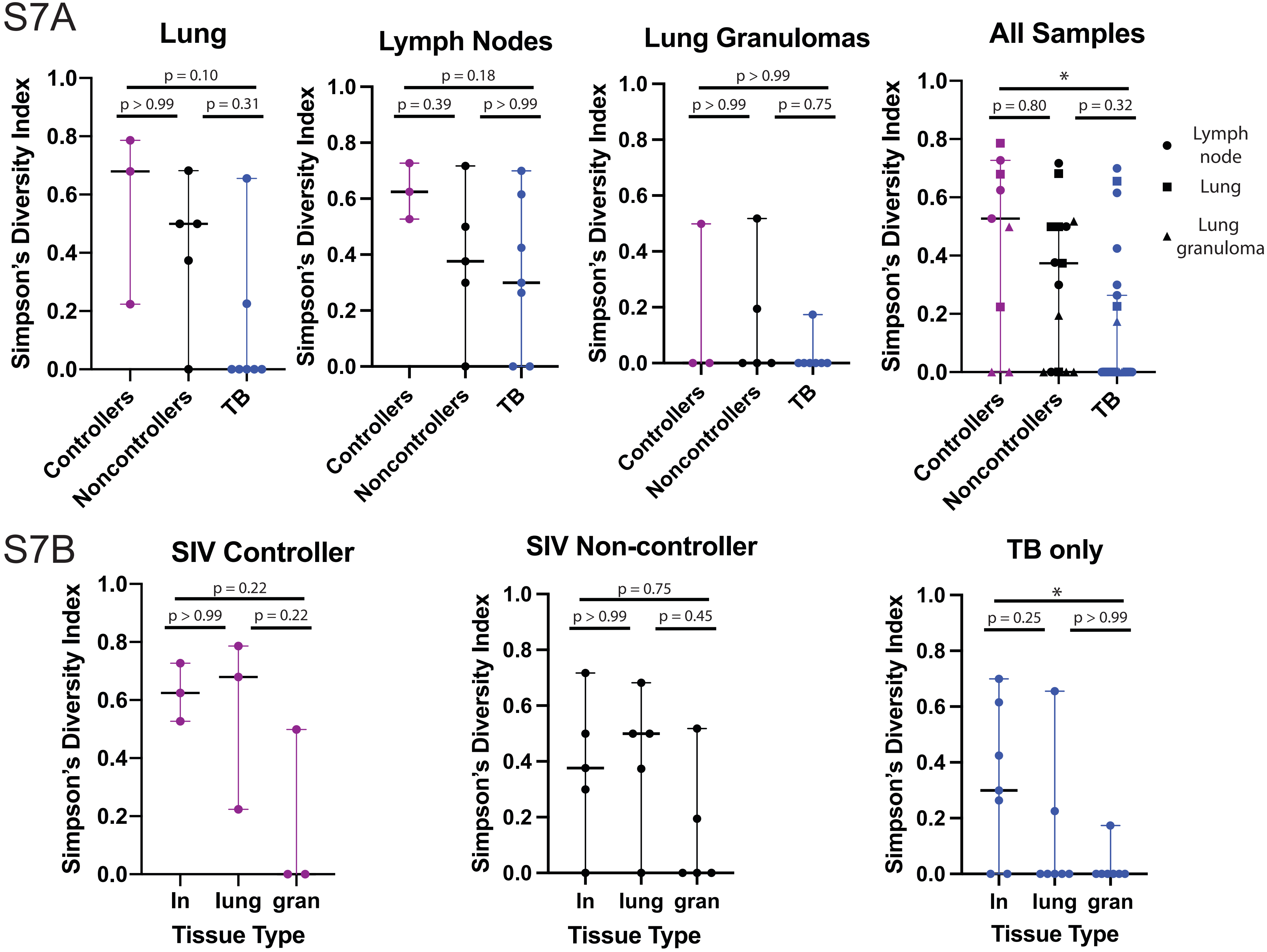
S7A
Lung
Lymph Nodes
Lung Granulomas
All Samples
Simpson's Diversity Index
p = 0.10
p > 0.99
p = 0.31
p = 0.18
p = 0.39
p > 0.99
p > 0.99
p > 0.99
p = 0.75
*
p = 0.80
p = 0.32
Controllers
Noncontrollers
TB
Lymph node
Lung
Lung granuloma
S7B
SIV Controller
SIV Non-controller
TB only
p = 0.22
p > 0.99
p = 0.22
p = 0.75
p > 0.99
p = 0.45
*
p = 0.25
p > 0.99
ln
lung
gran
Tissue Type

Supplemental Table 1

| Symbol | Animal | Infection | Controller status | MHC Haplotype |
|---|---|---|---|---|
| ● | 64-17 | SIV/TB | Controller | M1/M3 (M2 DP) |
| ■ | 65-17 | SIV/TB | Noncontroller | M1/M4 |
| ▲ | 66-17 | SIV/TB | Noncontroller | M1/M4 |
| ▼ | 73-17 | SIV/TB | Noncontroller | M1/M2 |
| ◆ | 256-17 | SIV/TB | Noncontroller | M1/M1 |
| □ | 258-17 | SIV/TB | Controller | M1/M3 |
| △ | 259-17 | SIV/TB | Noncontroller | M1/M4 |
| ▽ | 260-17 | SIV/TB | Controller | M1/M1 (recomb) |
| ▼ | 254-17 | TB | | M1/M4 |
| ○ | 255-17 | TB | | M1/M3 |
| □ | 257-17 | TB | | M1/M2 |
| ● | 62-17 | TB | | M1/M4 |
| ■ | 63-17 | TB | | M1/M4-6 |
| ▲ | 70-17 | TB | | M1/M1 class I, M1/M6 (M4M7DP) class II |
| ▽ | 81-18 | TB | | M1/M3 |
| △ | 84-18 | TB | | M1/M3 |

Supplemental Table 2

| Cohort | Animal | Number of Spleen granulomas | Number of Liver granulomas |
|---|---|---|---|
| SIV+ Viral Controller | 64-17 | 1-3 | 4-10 |
| | 258-17 | 0 | 0 |
| | 260-17 | 0 | 1-3 |
| SIV+ Viral Non-controller | 65-17 | 0 | 10-20 |
| | 66-17 | >50 | >50 |
| | 73-17 | 20-50 | 20-50 |
| | 256-17 | 0 | 0 |
| | 259-17 | >50 | >50 |
| SIV naïve | 62-17 | 4-10 | 10-20 |
| | 63-17 | 4-10 | 20-50 |
| | 70-17 | 0 | 1-3 |
| | 81-18 | 4-10 | >50 |
| | 84-18 | >50 | >50 |
| | 254-17 | 0 | 0 |
| | 255-17 | >50 | >200 |
| | 257-17 | 1-3 | 1-3 |

# Supplemental Table 3

| Name | Sequence | Scale | Purification |
|---|---|---|---|
| **Ftotal1** | CCCTACACGACGCTCTTCCGATCTNCNNNCNNNCNNNGCGCAACGCGTGCGG | 100nm | PAGE |
| **Ftotal2** | CCCTACACGACGCTCTTCCGATCTNNCNNNCNNNCNNNGCGCAACGCGTGCGG | 100nm | PAGE |
| **Ftotal3** | CCCTACACGACGCTCTTCCGATCTNNNCNNNCNNNCNNNGCGCAACGCGTGCGG | 100nm | PAGE |
| **Ftotal4** | CCCTACACGACGCTCTTCCGATCTCNNNCNNNCNNNGCGCAACGCGTGCGG | 100nm | PAGE |
| **Rtotal1** | GTGACTGGAGTTCAGACGTGTGCTCTTCCGATCTNGagaccacaacggtttccatatg | 100nm | PAGE |
| **Rtotal2** | GTGACTGGAGTTCAGACGTGTGCTCTTCCGATCTNNNGAgaccacaacggtttccatatg | 100nm | PAGE |
| **Rtotal3** | GTGACTGGAGTTCAGACGTGTGCTCTTCCGATCTNNNNGAgaccacaacggtttccatatg | 100nm | PAGE |
| **Rtotal4** | GTGACTGGAGTTCAGACGTGTGCTCTTCCGATCTGAgaccacaacggtttccatatg | 100nm | PAGE |
| **F501** | AATGATACGGCGACCACCGAGATCTACACTATAGCCTACACTCTTTCCCTACACGACGCTCTTC | 100nm | PAGE |
| **F502** | AATGATACGGCGACCACCGAGATCTACACATAGAGGCACACTCTTTCCCTACACGACGCTCTT | 100nm | PAGE |
| **F503** | AATGATACGGCGACCACCGAGATCTACACCCTATCCTACACTCTTTCCCTACACGACGCTCTTC | 100nm | PAGE |
| **F504** | AATGATACGGCGACCACCGAGATCTACACGGCTCTGAACACTCTTTCCCTACACGACGCTCTT | 100nm | PAGE |
| **F505** | AATGATACGGCGACCACCGAGATCTACACAGGCGAAGACACTCTTTCCCTACACGACGCTCTT | 100nm | PAGE |
| **F506** | AATGATACGGCGACCACCGAGATCTACACTAATCTTAACACTCTTTCCCTACACGACGCTCTTC | 100nm | PAGE |
| **F507** | AATGATACGGCGACCACCGAGATCTACACCAGGACGTACACTCTTTCCCTACACGACGCTCTT | 100nm | PAGE |
| **F508** | AATGATACGGCGACCACCGAGATCTACACGTACTGACACACTCTTTCCCTACACGACGCTCTTC | 100nm | PAGE |
| **R701** | CAAGCAGAAGACGGCATACGAGATCGAGTAATGTGACTGGAGTTCAGACGTGTGC | 100nm | PAGE |
| **R702** | CAAGCAGAAGACGGCATACGAGATTCTCCGGAGTGACTGGAGTTCAGACGTGTGC | 100nm | PAGE |
| **R703** | CAAGCAGAAGACGGCATACGAGATAATGAGCGGTGACTGGAGTTCAGACGTGTGC | 100nm | PAGE |
| **R704** | CAAGCAGAAGACGGCATACGAGATGGAATCTCGTGACTGGAGTTCAGACGTGTGC | 100nm | PAGE |
| **R705** | CAAGCAGAAGACGGCATACGAGATTTCTGAATGTGACTGGAGTTCAGACGTGTGC | 100nm | PAGE |
| **R706** | CAAGCAGAAGACGGCATACGAGATACGAATTCGTGACTGGAGTTCAGACGTGTGC | 100nm | PAGE |
| **R707** | CAAGCAGAAGACGGCATACGAGATAGCTTCAGGTGACTGGAGTTCAGACGTGTGC | 100nm | PAGE |
| **R708** | CAAGCAGAAGACGGCATACGAGATGCGCATTAGTGACTGGAGTTCAGACGTGTGC | 100nm | PAGE |
| **R709** | CAAGCAGAAGACGGCATACGAGATCATAGCCGGTGACTGGAGTTCAGACGTGTGC | 100nm | PAGE |
| **R710** | CAAGCAGAAGACGGCATACGAGATTTCGCGGAGTGACTGGAGTTCAGACGTGTGC | 100nm | PAGE |
| **R711** | CAAGCAGAAGACGGCATACGAGATGCGCGAGAGTGACTGGAGTTCAGACGTGTGC | 100nm | PAGE |
| **R712** | CAAGCAGAAGACGGCATACGAGATCTATCGCTGTGACTGGAGTTCAGACGTGTGC | 100nm | PAGE |

Figure S1. Dose Mtb Erdman CFU used for each animal by cohort. Each color/symbol combination indicates a unique animal. SIV+ viral controllers are shown in purple, SIV+ viral non-controllers are shown in black, and TB only (SIV-naïve) animals are shown in blue. Lines show median +/- 95% CI. Significance determined by Kruskal-Wallis with Dunn's test for multiple comparisons.

Figure S2. (A) Median tissue SIV copies/cell per animal for SIV+ viral controllers (purple) and SIV+ viral non-controllers (black). (B) Median tissue CFU per animal for SIV+ viral controllers (purple), SIV+ viral non-controllers (black), and TB only (SIV-naive) animals (blue). Lines represent median +/- 95% CI. Significance determined by Kruskal-Wallis with Dunn's test for multiple comparisons: *, $p < 0.05$, **, $p <0.01$, ***, $p < 0.001$, ****, $p < 0.0001$.

Figure S3. (A) Total CFU per animal and (B) percent sterile lung granulomas for each animal and cohort. Each animal indicated by a unique color/shape combination. Lines represent median +/- 95% CI. Significance determined by Kruskal-Wallis with Dunn's test for multiple comparisons.

Figure S4. Correlation of SIV present in individual lesions measured by copies/cell and bacterial CFU per CFU+ lesion in lung granulomas, thoracic LNs, and lung tissue for SIV+ viral non-controllers (black) and SIV+ viral controllers (purple). Spearman correlation coefficients were done to determine significance. Correlation was considered significant if $p < 0.05$.

Figure S5. Animal medians of total CD4+ (A, B) and total CD8+ (C,D) T cells producing TNF$\alpha$ (A, C) or IFN$\gamma$ (B, D) in the lung granulomas (left panel) or thoracic LN (right panel) in SIV+ viral controllers (purple), SIV+ viral non-controllers (black), and TB only (SIV-

naïve, blue) animals. Each dot represents a single animal. Lines represent median +/- 95% CI. Significance determined by Kruskal-Wallis with Dunn's test for multiple comparisons: *, $p < 0.05$, **, $p < 0.01$, ***, $p < 0.001$, ****, $p < 0.0001$.

Figure S6. Animal medians of total CD4+ (A, B) and total CD8+ (C,D) T cells expressing activation markers PD1 (A, C) or TIGIT (B, D) in the lung granulomas (left panel) or thoracic LN (right panel) in SIV+ viral controllers (purple), SIV+ viral non-controllers (black), and TB only (SIV-naïve, blue) animals. Each dot represents a single animal. Lines represent median +/- 95% CI. Significance determined by Kruskal-Wallis with Dunn's test for multiple comparisons: *, $p < 0.05$, **, $p < 0.01$, ***, $p < 0.001$, ****, $p < 0.0001$.

Figure S7. Animal medians of Mtb barcode diversity as calculated by Simpson's Diversity Index in (A, left to right) uninvolved lung, LN, granulomas, and all combined tissue samples, as well as (B) between SIV+ viral controllers (purple), SIV+ viral non-controllers (black), and TB only (SIV-naïve, blue) animals. Lines represent median +/- 95% CI. Significance is determined by Kruskal-Wallis with Dunn's test for multiple comparisons: *, $p < 0.05$, **, $p < 0.01$, ***, $p < 0.001$, ****, $p < 0.0001$

Supplemental Table 1. Table describing animals used in this study, the symbol used to identify each, the infection group, whether or not the SIV+ animals spontaneously controlled SIV replication or not, and their MHC haplotype.

Supplemental Table 2. Number of granulomas present in the spleen and liver in each animal at necropsy.

Supplemental Table 3. Primer names, sequences, scale, and purification used in Mtb barcode sequencing.
